# A Proposed Approach for the Management of Clot-in-Transit

**DOI:** 10.7759/cureus.28481

**Published:** 2022-08-27

**Authors:** Aayushi N Patel, Rahulkumar J Amrutiya, Buddhadev N Manvar

**Affiliations:** 1 Clinical Research, Tampa General Hospital, Tampa, USA; 2 Cardiology, St. Jude Medical Center, New York City, USA; 3 Cardiology, The Brooklyn Hospital Center, New York City, USA

**Keywords:** surgical thrombectomy (st), anticoagulant therapy, angiovac system, catheter-directed thrombolysis, free floating right heart thrombus, right heart clot, pulmonary embolism (pe), point of care ultrasound (pocus), thrombus in transit, clot in transit

## Abstract

Clot-in-transit (CIT) is defined as a mobile echogenic material in the right atrium or ventricle as observed on ultrasound. A right heart free-floating thrombus is unusual when there is no structural disease of the heart or atrial fibrillation. Cardiopulmonary collapse and quick death can come from CIT, which occurs when a blood clot moves from the heart to the lungs. There are some clinical case reports of a large volume thrombus that was freely floating in the right heart in an asymptomatic patient, and the best therapeutic options are uncertain. Although several trials have been conducted on the treatment of CIT, clinical judgment is still used to determine the best treatment for right heart thrombus (RHT), especially when associated with pulmonary embolism (PE). In this review article, we discuss various diagnostic modalities and treatment options for this rare malady. We studied in detail their clinical impact on patients according to past research studies.

## Introduction and background

Clot-in-transit (CIT) refers to a noticeable, free-floating thrombus inside the right-side chambers of the heart, although a thrombus in the superior vena cava (SVC) or inferior vena cava (IVC) may also fall under this heading. This type of clot is at high risk of embolization into the pulmonary artery. A right-sided heart clot is unusual, especially in the absence of a structural heart defect, in-situ catheter, or atrial fibrillation. It can be found at the bedside when we perform emergent echocardiography to assess dysfunction of the right ventricle. About 4% of patients suffering from a pulmonary embolism (PE) have a floating embolus present in the right heart, a finding that is associated with a mortality rate of more than 40% [1,2]. So, PE coupled with CIT has a higher mortality rate. Because of its high mortality rate, which ranges from 21% in 14 days to 29% in 3 months, a right-side CIT is considered a medical emergency [1]. The therapeutic options include thrombolysis, anticoagulation, catheter-directed thrombolysis, and surgical thrombectomy. The best treatment option is unknown [2,3,4].

## Review

We reviewed all the articles from the year 1982 to the present for similar studies. We used the keywords “clot in transit”, “thrombus in transit”, “treatment of the clot in transit”, “treatment of thrombus in transit”, “pulmonary embolism”, “anticoagulation”, and “Point-of-care Ultrasound (POCUS)” to find similar publications on PubMed, Google Scholar, *Chest *journal and *The American Journal of Medicine*. We screened the titles and abstracts of various studies to disregard the non-relevant studies such as those associated with coronavirus disease 2019 (COVID-19) patients and articles published in a language other than English. Inclusion criteria were the following: full-text articles published within the last 40 years, observational studies, case reports, systematic reviews, meta-analyses, narratives, and articles specifically on the diagnosis and management of CIT. Gender differences were not taken into account. We observed that there is a lack of interventional studies to favor one form of treatment strongly over the others.

Role of imaging modalities

Echocardiography

Echocardiography is still the gold standard for pulmonary embolism risk classification and detection of intracardiac thrombi, with the added benefit of determining the clot's origin. Right heart clots can be classified into three categories based on their morphology:

Type A: Type A is the most common type. It occurs when a deep vein thrombus (DVT) breaks free and floats up to the right side of the heart. It carries the greatest risk of embolization into the pulmonary circuit. It moves freely inside the heart chambers and has an elongated, wormlike form.

Type B: The atrium or ventricle is assumed to be the source of a Type B thrombus. It is oval, has a broad base, and is adhered to the chamber wall.

Type C: Type C thrombi are uncommon, extremely migratory clots that look like cardiac myxomas [5].

Echocardiography can also be used to track right heart function after thrombolysis.

The presence of thrombi in the right heart can easily be misinterpreted as other physiological or pathological signs. One should differentiate thrombi in the right heart from congenital structures such as the atrial septal aneurysms, persistent eustachian or thebesian valves, Chiari network, and acquired pathologies such as intracardiac malignancies and vegetations [6].

If CIT is associated with acute PE (a high mortality combination), then echocardiography will show the following evidence of right heart strain: interventricular septum bowing into the left ventricle (LV), systolic dysfunction of the right ventricle (RV), and McConnell’s sign (akinesis of the free wall of the right ventricle with sparing of the apex) [7].

When transthoracic echocardiography (TTE) is non-diagnostic, transoesophageal echocardiography (TEE) is utilized to obtain a better image of the thrombus. While performing thrombectomy, real-time monitoring of the clot during atrial entry can be done using TEE. Such surveillance can decrease the possibility of collateral damage, including rupture of chordae tendineae and subsequent arrhythmia incitement. TEE monitoring also lessens the requirement for fluoroscopy, which lowers the dangers of ionizing radiation and contrast injection [8].

An invasive imaging technique called intravascular ultrasound (IVUS) can precisely detect CIT both before and during an embolectomy [9]. Another invasive imaging modality is intracardiac ultrasonography (ICE). Both of these techniques could end up being more useful. Because ICE only requires one operator, interventional cardiologists frequently use it compared to IVUS. This removes the requirement of TEE, which circumvents the possibility of tracheal trauma. Additionally, ICE cuts down on procedural and fluoroscopy times [10]. In their case report, Chen et al. documented a successful right atrial thrombus aspiration that was guided by ICE [11].Likewise, IVUS is used while performing embolectomy [9]. The limited availability and higher cost of IVUS and ICE may prevent them from being used as much as TTE or TEE, which are more widely available.

Various modalities can be used to diagnose CIT, such as echocardiography (POCUS, TTE, or TEE), Computed Tomography (CT), and Computed Tomography Angiography (CTA). The sensitivity and specificity of various imaging modalities for intracardiac thrombi have been presented in Table 1. Yastrebov et al. stated that 3-Dimensional (3D) intracardiac echocardiography gives a precise and better picture of the heart’s anatomy and pathology compared to 2-Dimensional (2D) echoes. It offers the benefit of diagnosing CIT and also providing accurate details like exact location, size, and relation to nearby structures [12]. This additional information facilitates the selection of an appropriate treatment plan. IVC, Right atrium (RA), and RV will show the presence of a filling defect on CTA if there is the presence of a CIT [13]. Timely and accurate diagnosis is so crucial for these patients. Gregory et al. report the case of a 71-year-old female with atrial fibrillation who was diagnosed with CIT by using POCUS immediately on arrival to the Emergency Department (ED). Because of the early diagnosis, the staff was already prepared for what was expected [14].

**Table 1:** Sensitivity and specificity of various imaging modalities to detect intracardiac thrombi

Imaging study	Sensitivity	Specificity
Transthoracic echocardiography	95%	86% [15]
Computed tomography	81%	90% [16]
Computed tomography angiography	100%	91-100% [17]

The prevalence of CIT is estimated to be between 4 and 18 percent; however, as POCUS becomes more prevalent, this number is expected to climb. The lack of a universal treatment guideline is to blame for a 100% mortality rate if left untreated [18]. So far, we can only rely on clinical acumen and detecting CIT early by using contemporary medical technologies.

Treatment

Catheter-Based Thrombolysis

High-frequency ultrasound exposure near the clot's surface or percutaneous catheter-directed thrombolysis, endovascular mechanical thrombectomy using a capture device (such as a basket) with fragmentation, as well as endovascular suction of the clot directly from the pulmonary arteries, ventricle, or atrium are just a few examples of approaches to intervention.

Advantage: These operations provide a high success rate and reduce the severity of major and minor bleeding [19].

Disadvantage: Risk of thrombus dislodgement; bulky thrombi are usually resistant to thrombolysis.

Two thrombectomy systems are presented in Table 2.

**Table 2:** Thrombectomy systems ECMO: extracorporeal membrane oxygenation; RV: right ventricle; CIT: clot-in-transit

Device Name	Description	Comments	Literature search
FlowTriever device	A mechanical and suction device.	Its lack of need for extracorporeal filtration or cardiopulmonary bypass is an advantage.	Few case reports have shown good results for treating CIT [19,20]
AngioVac device	A thrombectomy tool with FDA approval can aspirate intravascular debris, including tumors, foreign substances, and thrombus.	The device's rigidity, mobility, and the possibility of RV perforation are major technical challenges. It can’t be used in noncardiovascular centers because it requires an ECMO setup and a perfusionist who isn't always accessible.	

In a literature review, Worku B. et al stated that AngioVac was an effective alternative to performing surgical thrombectomy for patients who presented with intracardiac or iliocaval thrombi with a success rate of more than 80% [21]. On the other hand, Bayona M et al. proposed that using FlowTriever would be better than using AngioVac due to no need for extracorporeal perfusion. It can be a preferred method for the treatment of CIT and PE, especially for people in whom thrombolysis is contraindicated [22].

Type A thrombus has a high risk for embolization, which is why they must be treated aggressively with thrombolytics and surgical embolectomy. Type B is at low risk for embolization and can be medically managed. Whereas for Type C, there is no strong literature supporting treatment, but Reddy et al. used Generation 3 AngioVac system to remove a Type C right atrial thrombi in a 25-year-old male patient who was in remission for acute myeloid leukemia. The patient had denied surgical thrombectomy and the systemic coagulation was failing to show any improvements [23]. When the clot was removed it was noticed that it was calcified, but AngioVac is used to remove soft, fresh clots [24].

Akhmerov A et al. discussed a retrospective study with 13 patients treated with AngioVac with variable indications and they found that 77% of patients made it to discharge from the hospital. Eight patients were suffering from right ventricular dysfunction, but after the procedure, 11 out of the 13 patients had markedly improved RV functions [25]. As we know that CIT causes right ventricular dysfunction, especially when associated with PE [26], AngioVac can serve as an extraordinary treatment not only to remove CIT but also to reduce the complications from CIT. But in a project by Rose et al., 177 cases of right heart thromboemboli were studied. Subjects were categorized into four different groups respective to the treatment modality and compared with mortality rates. Mortality was 100% without any treatment, 28.6% with anticoagulation, 23.8% with surgical embolectomy, and 11.3% with thrombolysis [27]. Given the lowest mortality with thrombolysis, it seems a great option but its use is concerning in patients with a bleeding disorder or history of head trauma. So, a trial for AngioVac can be performed as an alternative in those patients. We propose that AngioVac would serve as a breakthrough model for the treatment of all types of CIT.

In an interventional study performed by Liu B et al. on 20 patients with DVT and acute PE, it was found that a combination of catheter-directed thrombolysis (CDT) and percutaneous mechanical thrombectomy (PMT) had a success rate of 100%. During their hospitalization, there were no drastic events. Only four patients had to undergo iliac vein stent placement due to iliac vein compression syndrome. The patients were followed up and no event of recurrence was noticed for 16.5+/-6.8 months [28]. Interventional studies should be performed adopting a similar strategy for a CIT as well.

Surgical Embolectomy

Depending on the size of the CIT, more invasive procedures may be required. Yang et al demonstrate a case report where they performed a surgical resection by lower mini-sternotomy due to the large size of the CIT [29]. It consists of surgically opening the right atria and/or pulmonary trunk and manually extracting the thrombus. It's coupled with a full cardiopulmonary bypass.

Advantage: If right-to-left heart communication is present, surgical embolectomy provides a more decisive treatment and may provide a chance to close the communication in the same sitting. For patients who are hemodynamically unstable, it is a favored treatment option [27,30].

Disadvantage: It necessitates substantial heart surgery as well as cardiopulmonary bypass. The technique cannot be performed in hospitals with minimal surgical infrastructure. Prolonged time-to-treatment, general anesthesia, cardiac bypass, secondary infection, and failure to eradicate concurrent pulmonary emboli outside the central pulmonary arteries are all possible risks [31,32,33]. 

Surgical Embolectomy in the Setting of Concomitant CIT and PE

Numerous studies showed that most of the time CIT was diagnosed when the patient presented with signs and symptoms suggestive of PE. Patients must be treated for CIT and PE simultaneously to avoid another episode of PE following CIT. According to a meta-analysis performed by selecting six cohort studies that consisted of 15,220 patients who suffered from symptomatic PE that was acute in onset and also had a right heart thrombus that was detected by echo, it was proved that there was a 3.0 times threat of poor short-term prognosis in patients having right heart thrombi (RHT) along with PE than PE alone [34]. Not only does it increase mortality, but it also changes the treatment plan. In a case report by Medina MA et al. of CT Scan diagnosed PE along with thrombus of the left subclavian artery, emergent surgical pulmonary embolectomy was performed for CIT with acute massive pulmonary embolism. They found that the technique is associated with lower risks and has excellent results [35].

Anticoagulants: The advantage of anticoagulants is the ease and rapidity of administration. They can be reserved for patients in whom surgery is contraindicated (e.g., advanced age, progressive cancer, recent brain surgery, gynecological-obstetric bleeding, and stroke). The disadvantage is that pulmonary or systemic ischemia events may occur as a result of bleeding problems or thrombus fragmentation. They only prevent clot proliferation; they do not affect a pre-existing clot.

Anticoagulants in the Setting of Concomitant CIT** **and PE

An evidence-based study was performed by Barrios et al. on 325 patients having RHT and PE. Patients received anticoagulation or anticoagulation + reperfusion. They found that there was not much statistical difference between both groups with regard to all-cause mortality and episodes of major bleeding. But in the follow-up period, it was found that 6.2% had a recurrence in the reperfusion group [36]. A case report by Mardinger demonstrated a report of a 32-year-old female suffering from a CIT and a PE postoperatively, who was successfully treated with only half a dose of anticoagulation in combination with intravenous (IV) unfractionated heparin [37].

Systemic Thrombolysis: The advantages are - it improves left and right ventricular output by increasing right ventricular function and minimizing RV-LV dependency. It also reduces pulmonary hypertension and improves thrombus lysis and pulmonary reperfusion [38]. Furthermore, it has the ability to dissolve clots in three separate places simultaneously: pulmonary embolus, intracardiac thrombus, and venous thrombosis. Lastly, it’s a straightforward, quick, and universally applicable intervention that can be performed at the patient's bedside [39]. Additionally, in a crashing, near-to-death patient, or during cardiac arrest, this may be the only option. The disadvantage is that there is a risk of embolizing in the lungs after the clot breaks free, especially when there is a thrombus already present in the lungs and bleeding.

**Figure 1:**
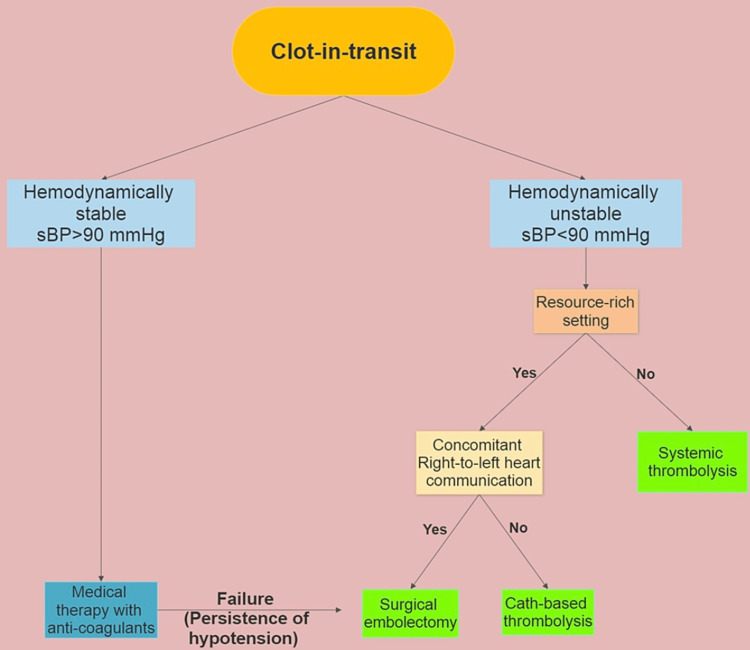
Suggested algorithm for the treatment of clot-in-transit Resource-rich setting: An operating room with cardiopulmonary bypass setup and experienced surgeon available round-the-clock.

Management plans should be undertaken based on individual cases until more definitive data is available, considering complicating conditions such as hemodynamic instability, right heart function, Patent Foramen Ovale (PFO), and malignancy. No randomized controlled trials have directly compared the therapy modalities; hence, the best therapeutic approach is still up for debate. We propose here an algorithm that depicts our approach for the treatment of CIT (Figure 1).

## Conclusions

The material reviewed in this paper focuses on the diagnosis and treatment of CIT. A CIT in the setting of PE is a potentially fatal illness that necessitates the involvement of a multidisciplinary team for prompt identification and treatment. 3D echo provides the highest accuracy in detecting CIT but due to lack of availability in most institutions, 2D echo serves as the best practical option as of now. The best treatment for CIT with a concomitant heart defect or with concomitant PE is surgical thrombectomy. Whereas for isolated CIT, catheter-based thrombectomy has shown superior results. The treatment modality should be adjusted according to the patient's comorbid diseases, and the treating center’s expertise should also be used to determine the best course of action. Patients should be risk-stratified so that the benefits in terms of mortality outweigh the risk of bleeding. More research is needed to build a tool that may consider characteristics including age, heart rate, respiratory rate, oxygen requirement, biomarkers, electrocardiogram, and RV dysfunction to establish the best therapeutic approach for CIT. This is a review article for busy physicians to have a cumulative view of managing CIT.

## References

[1] Torbicki A, Galié N, Covezzoli A, Rossi E, De Rosa M, Goldhaber SZ (2003). Right heart thrombi in pulmonary embolism: results from the International Cooperative Pulmonary Embolism Registry. J Am Coll Cardiol.

[2] Chartier L, Béra J, Delomez M (1999). Free-floating thrombi in the right heart: diagnosis, management, and prognostic indexes in 38 consecutive patients. Circulation.

[3] Chapoutot L, Nazeyrollas P, Metz D, Maes D, Maillier B, Jennesseaux C, Elaerts J (1996). Floating right heart thrombi and pulmonary embolism: diagnosis, outcome and therapeutic management. Cardiology.

[4] Dalen JE (2017). Free-floating right heart thrombi. Am J Med.

[5] (1989). The European Cooperative Study on the clinical significance of right heart thrombi. European Working Group on Echocardiography. Eur Heart J.

[6] Ragland MM, Tak T (2006). The role of echocardiography in diagnosing space-occupying lesions of the heart. Clin Med Res.

[7] McConnell MV, Solomon SD, Rayan ME, Come PC, Goldhaber SZ, Lee RT (1996). Regional right ventricular dysfunction detected by echocardiography in acute pulmonary embolism. Am J Cardiol.

[8] Miller SD, Lee DC, Dollar BT, Schepel SR, Shestopalov A, Culp WC Jr (2019). Transesophageal echocardiography guidance for atrial-caval thrombus removal with the AngioVac system. Proc (Bayl Univ Med Cent).

[9] Cornman-Homonoff J, Kishore S, Camacho JC, Winokur RS (2019). Intravascular Ultrasound-Guided Extraction of Free-Floating Inferior Vena Cava Tumor Thrombus Using the ClotTriever Mechanical Thrombectomy Device. J Vasc Interv Radiol.

[10] Enriquez A, Saenz LC, Rosso R, Silvestry FE, Callans D, Marchlinski FE, Garcia F (2018). Working with the anatomy rather than fighting it. Circulation.

[11] Chen D, Sachdeva R, Kumar G (2021). Right Atrial Thrombus in Transit. Cath Lab Digest.

[12] Yastrebov K, Brunel L, Paterson HS, Williams ZA, Bannon PG (2020). Three-dimensional intracardiac echocardiography and pulmonary embolism. Cardiovasc Ultrasound.

[13] Khosla A, Mojibian H, Assi R, Tantawy H, Singh I, Pollak J (2022). Right heart thrombi (RHT) and clot in transit with concomitant PE management: approach and considerations. Pulm Circ.

[14] Gregory D, Becker BA, Collin M (2021). Elderly woman with shortness of breath. J Am Coll Emerg Physicians Open.

[15] Stratton JR, Lighty GW Jr, Pearlman AS, Ritchie JL (1982). Detection of left ventricular thrombus by two-dimensional echocardiography: sensitivity, specificity, and causes of uncertainty. Circulation.

[16] Wu X, Wang C, Zhang C, Zhang Y, Ding F, Yan J (2012). Computed tomography for detecting left atrial thrombus: a meta-analysis. Arch Med Sci.

[17] Groeneveld NS, Guglielmi V, Leeflang MM (2020). CT angiography vs echocardiography for detection of cardiac thrombi in ischemic stroke: a systematic review and meta-analysis. J Neurol.

[18] Puello F, Harewood J, Lee C, Rupani N, Abe O (2017). Thrombus in transit: the emergence of a deadly diagnosis. Chest.

[19] Kuo WT, Gould MK, Louie JD, Rosenberg JK, Sze DY, Hofmann LV (2009). Catheter-directed therapy for the treatment of massive pulmonary embolism: systematic review and meta-analysis of modern techniques. J Vasc Interv Radiol.

[20] Nezami N, Chockalingam A, Cornman-Homonoff J, Marino A, Pollak J, Mojibian H (2020). Mechanical thrombectomy for pulmonary embolism in patients with patent foramen Ovale. CVIR Endovascular.

[21] Worku B, Salemi A, DʼAyala MD, Tranbaugh RF, Girardi LN, Gulkarov IM (2016). The Angiovac device: understanding the failures on the road to success. Innovations.

[22] Bayona Molano MD, Salsamendi J, Mani N (2021). Emergent mechanical thrombectomy for right atrial clot and massive pulmonary embolism using flowtriever. Clin Case Rep.

[23] Reddy PKV, Kwan T, Latouff O, Patel A (2021). Suction thrombectomy of a massive, hypermobile (type C) right atrial thrombus: a case report. Eur Heart J Case Rep.

[24] (2022). Angiovac | Florida Electrophysiology Associates. https://heartbeatdoctor.com/procedures/angiovac/.

[25] Akhmerov A, Reich H, Mirocha J, Ramzy D (2019). Effect of percutaneous suction thromboembolectomy on improved right ventricular function. Tex Heart Inst J.

[26] Khanna S, Mehta A (2018). Images in anesthesiology: a clot in transit: impending paradoxical embolization. Anesthesiology.

[27] Rose PS, Punjabi NM, Pearse DB (2002). Treatment of right heart thromboemboli. Chest.

[28] Liu B, Liu M, Yan L, Yan J, Wu J, Jiao X, Guo M (2018). Percutaneous mechanical thrombectomy combined with catheter-directed thrombolysis in the treatment of acute pulmonary embolism and lower extremity deep venous thrombosis: a novel one-stop endovascular strategy. J Int Med Res.

[29] Yang YC, Chen YY (2021). Right heart thrombus-in-transit in a patient with Evans syndrome: a case report. Medicine (Baltimore).

[30] Huwer H, Winning J, Isringhaus H, Kalweit G (2004). Transit thrombus entrapped in a patent foramen ovale. Heart Lung.

[31] Claver E, Larrousse E, Bernal E, López-Ayerbe J, Valle V (2004). Giant thrombus trapped in foramen ovale with pulmonary embolus and stroke. J Am Soc Echocardiogr.

[32] Maroto LC, Molina L, Carrascal Y, Rufilanchas JJ (1997). Intracardiac thrombus trapped in a patent foramen ovale. Eur J Cardiothorac Surg.

[33] Can I, Altunkeser BB, Yavas O, Duzenli A, Ozdemir K, Gok H (2006). Transit thrombus entrapped in patent foramen ovale resolved without clinical embolic events. J Am Soc Echocardiogr.

[34] Barrios D, Rosa-Salazar V, Morillo R (2017). Prognostic significance of right heart thrombi in patients with acute symptomatic pulmonary embolism. Chest.

[35] Medina MA, Guerrero AF, Sandoval NF, Umana JP (2020). Successful surgical treatment of clot in transit with impending paradoxical embolism: a case report. JTCVS Tech.

[36] Barrios D, Chavant J, Jiménez D (2017). Treatment of right heart thrombi associated with acute pulmonary embolism. Am J Med.

[37] Mardinger C, Boiteau PJ, Kortbeek JB (2020). Thrombolysis of postoperative acute pulmonary embolism with a thrombus in transit. Case Rep Med.

[38] Jardin F, Dubourg O, Guéret P, Delorme G, Bourdarias JP (1987). Quantitative two-dimensional echocardiography in massive pulmonary embolism: emphasis on ventricular interdependence and leftward septal displacement. J Am Coll Cardiol.

[39] Ferrari E, Benhamou M, Berthier F, Baudouy M (2005). Mobile thrombi of the right heart in pulmonary embolism: delayed disappearance after thrombolytic treatment. Chest.

